# Case Report: A novel intergenic MIR4299/MIR8070-RET fusion with RET amplification and clinical response to pralsetinib in a lung adenocarcinoma patient

**DOI:** 10.3389/fonc.2022.929763

**Published:** 2022-09-26

**Authors:** Sha-Sha Wang, Fang Wang, Zhen Zeng, Fang Gao, Huan-Huan Liu, Hui-Na Wang, Yi Hu, Hai-Feng Qin

**Affiliations:** ^1^ Department of Oncology, Chinese People's Liberation Army General Hospital, Beijing, China; ^2^ Department of Internal Medicine, OASIS International Hospital, Beijing, China; ^3^ Department of Medicine, Acornmed Biotechnology Co., Ltd., Beijing, China

**Keywords:** RET intergenic fusion, pralsetinib, lung adenocarcinoma, novel intergenic, tyrosine kinase inhibitors

## Abstract

The identification of receptor-tyrosine kinase gene (RET) fusions in lung cancer has become crucial owing to actionable events that predict responsiveness to tyrosine kinase inhibitors (TKIs). However, RET fusions with distinct partner genes respond differently to TKIs. In this case, a 60-year-old man was diagnosed with advanced lung adenocarcinoma. A novel RET-MIR4299/MIR8070 fusion and RET amplification were identified using next-generation sequencing (NGS). The patient was then administered with pralsetinib. After 3 weeks of therapy, the patient had a partial response. At the time of reporting, the patient was on continuous pralsetinib. These findings broaden the range of RET fusion types and provide the basis for the hypothesis that RET intergenic fusion and amplification respond to pralsetinib treatment in lung adenocarcinoma.

## Introduction

Receptor-tyrosine kinase gene (RET) fusion occurs in 1.4% of non-small cell lung cancer (NSCLC) and 1.7% of lung adenocarcinoma (1) in China. Patients harboring RET fusions have improved outcomes with the advent of RET inhibitors. It has been reported that the distinct type of RET fusion partner has a different response to the tyrosine kinase inhibitor (TKI) (2). The most common fusion partners of RET described are KIF5B and CCDC6 (3). In a recent study, 137 Chinese lung cancer patients with RET fusion were found, and most common partner genes were KIF5B (62%) and CCDC6 (21%), with the novel RET fusions accounting for 12.4% (4). It is necessary to investigate RET fusion partners and their qualifications for RET-based targeted therapy.

Pralsetinib is a selective TKI with anticancer activity (5). However, the response of pralsetinib to novel RET fusions remains unknown. Here, we report a patient with lung adenocarcinoma who harbored a novel MIR4299/MIR8070-RET fusion with RET amplification and responded to pralsetinib.

## Case representation

**Figure 1:**
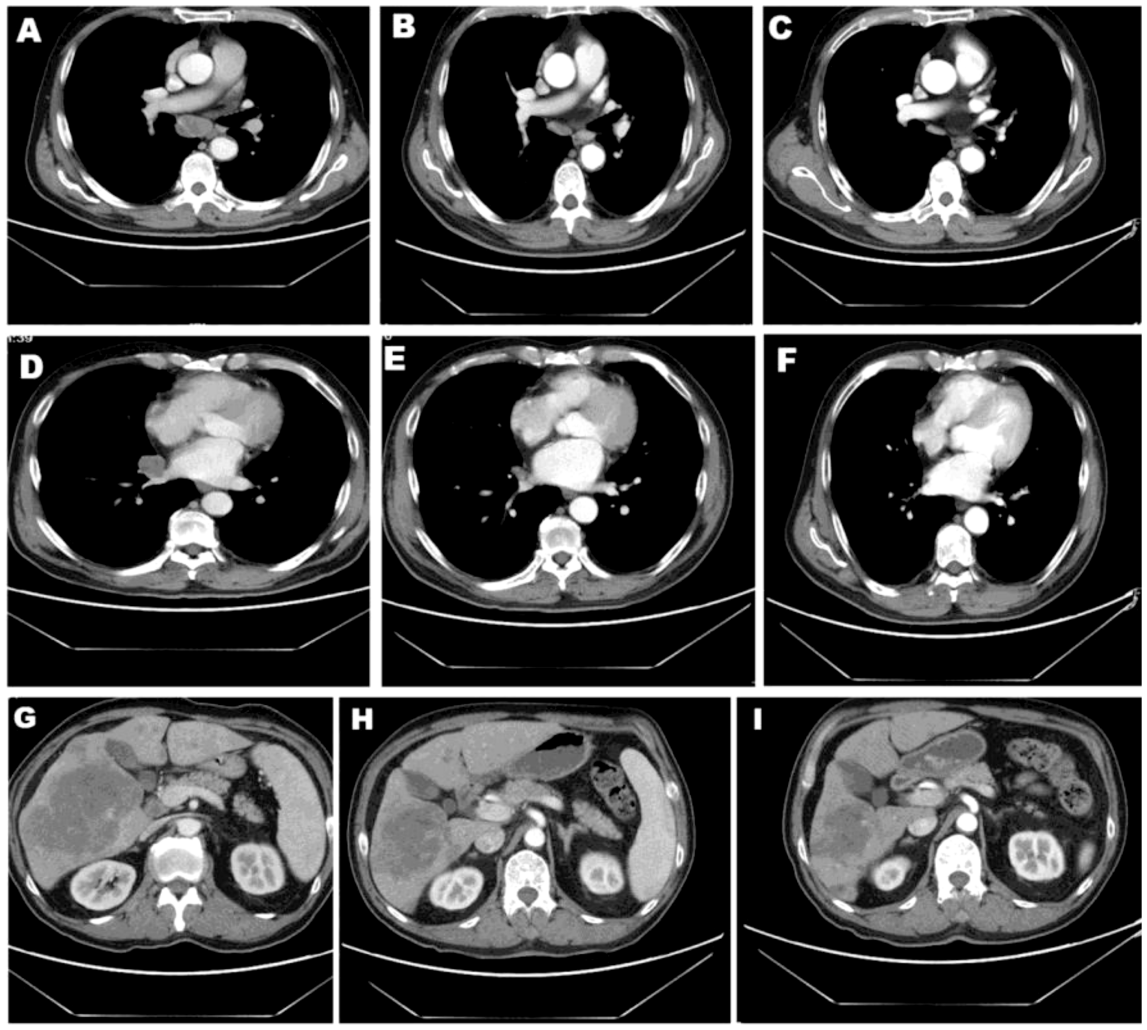
Patient CT images. CT scans of lesion 1 **(A–C)** and lesion 2 **(D–F)** in lung as well as lesion in liver **(G–I)** before and after 1 or 2 months of pralsetinib treatment.

**Figure 2:**
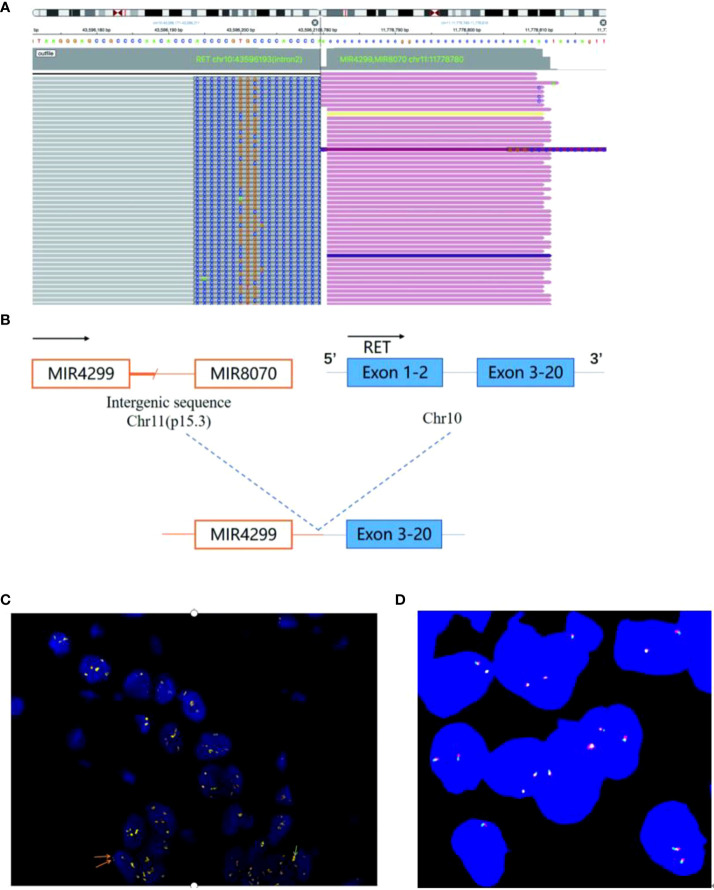
NGS and FISH findings. **(A)** NGS analysis showing a breakpoint of the MIR4299/MIR8070-RET fusion and their sequence information. **(B)** Schematic diagram of the MIR4299/MIR8070-RET fusion. **(C)** FISH results showing the RET signal. FISH tests on lung biopsy tissue from the patient revealed more than 60% tumor content. FISH with RET dual color break-apart probe. The red signal represents the 5’ end RET gene while the green signal represents the 3’ end RET gene. The signal separated red and green signals, consistent with RET fusion. In most cases, the red signal and green signal were synchronized in clusters, and RET gene amplification could be confirmed by combining microscopic observation. Yellow represents red and green signals superimposed. In addition, the amplified signal (yellow signal, or clusters of red-green signal) is too strong, which obscures the fusion signal. **(D)** The image of negative control showing each color probe without fusion gene or amplification.

A 60-year-old male nonsmoker was admitted with nausea, fatigue, and mulligrubs after meal. Nodules of liver were detected by ultrasonography. Computed tomography (CT) scan revealed two space-occupying lesions in the lower lobe of the right lung and diffuse hepatic metastases (**Figures 1A, G**). The pathological diagnosis was stage IV lung adenocarcinoma. The circulating tumor DNA (ctDNA) of the patient was analyzed using next-generation sequencing (NGS) to detect 808 tumor-associated genes (**supplementary file**), including all clinically relevant biomarkers for NSCLC. NGS identified a novel MIR4299/MIR8070-RET fusion with RET amplification. The abundance of fusion was 51.49%, and the copy number variation (CNV) number was 19.6 (**Figures 2A, B** and **Table 1**). Fluorescence *in situ* hybridization (FISH) further confirmed the RET fusion and amplification (**Figure 2C**). The fusion encompassed MIR4299/MIR8070 intergenic region and exons 3 to 20 of RET, holding the whole RET kinase domain, which has never been reported before. RT-PCR was not performed since the tumor tissues were immersed in formalin, which caused serious degradation of RNA, but this degradation did not affect FISH results (6–8). NGS and immunohistochemistry (IHC) assay showed that TMB was 13.08 mut/Mb, and programmed death-ligand 1 (PD-L1) expression was less than 1%. He was subsequently treated with pralsetinib (400 mg orally once daily). After a month, the CT findings showed shrinkage of the lung lesions by 36.11% and 37.5% (**Figures 1A, B, D, E**) and reduction of the liver lesions by 18.68% (**Figures 1G, H**). Meanwhile, the levels of carcino-embryonic antigen (CEA) (from 23,385 to 8,664 ng/ml), progastrin-releasing peptide (from 41,206 to 7,482 pg/ml), and tissue polypeptide specific antigen (from 4,500 to 141.9 U/L) were markedly reduced. Therefore, he was considered to achieve a partial response (PR) according to RECIST 1.1 criteria and continued to receive pralsetinib (400 mg orally once daily). One month later, the decreases in tumor size of 13.04% and 6.7% in lung as well as 21.62% in liver were shown on CT (**Figures 1C, F, I**). At the time of reporting, the patient was still undergoing pralsetinib treatment.

**Table 1:** Mutations detected by NGS in this patient.

Gene	Exon	Mutation	Abundance (%)	CNV number
TP53	exon7	p.R249S	89.96	–
NOTCH1	exon8	p.G427fs	92.13	–
KMT2D	exon34	p.E3090X	39.42	–
HNF1A	exon2	p.Y166X	52.23	–
SMARCA4	exon21	p.E1023X	93.13	–
LRP1B	Exon78	p.R3972G	34.6	–
RET	–	CNV	–	19.6
KDM5A	–	CNV	–	4.0
EGFR	–	CNV	–	4.5
RET	E2:E-	RET-MIR4299, MIR8070	51.49	–

CNV, copy number variation.

## Discussion

As the technology for ctDNA has advanced, NGS detection of fusion using ctDNA has become feasible (9, 10). NGS displays greater strengths in identifying a fusion variant than IHC or FISH since it provides specific partner genes and fusion breakpoints. As a result of NGS and FISH analysis, we confirmed that the patient harbored a novel RET intergenic-breakpoint MIR4299/MIR8070 fusion (abundance of 51.49%), as well as coexisting RET amplification (CNV number of 19.6) and responded to pralsetinib treatment.

To our knowledge, this is the first case harboring a novel RET intergenic-breakpoint MIR4299/MIR8070 fusion with coexisting RET amplification and responding to pralsetinib treatment. Pralsetinib is a selective tyrosine kinase inhibitor (TKI) approved for the treatment of NSCLC with RET fusion and the overall response rate (ORR) is 61% regardless of RET fusion partner (11).

In the treatment of NSCLC, RET-target therapy has shown exceptional results. Noteworthily, distinct RET fusion variants’ reactions to TKIs were observed to be heterogeneous. Sun et al. found that a new MYH9-RET fusion developed resistance to simertinib therapy (12). In contrast, Montrone and colleagues reported that a patient with advanced lung adenocarcinoma with RET fusion was treated with pralsetinib and had an outstanding clinical and radiological response as well as good tolerability (13), which is similar with our patient. RET amplification showed similar response when treated with vandetanib and placebo in a phase III NSCLC clinical trial (14). However, Paratala et al. reported that RET amplification can induce transformation of non-tumorigenic cells, support xenograft tumor formation, and render sensitivity to RET inhibition in breast cancer (15). Based on these results, we hypothesized that the combination of RET fusion and amplification enhanced the response to TKIs. Since MIR4299 was highly expressed in normal cells (16), we speculated that MIR4299 expression and function in normal cells may drive RET gene amplification in tumor cells with the RET-MIR4299 fusion gene. There is a possibility that RET intergenic-breakpoint MIR4299/MIR8070 fusion genes within episomes integrate into chromosomes but then amplify. The proposed hypothesis for how this amplification occurs is that the integration occurs downstream of a strong promoter (16, 17). Future studies will be needed to see whether high RET amplification represents important association to sensitivity to RET fusion-targeted therapies. This patient had a novel intergenic RET fusion plus RET amplification and achieved PR within 1 month. RET activation may contribute to a series of oncogenic signaling pathways, resulting in tumorigenesis and metastasis (3). Only fusion variants that maintain the complete RET kinase domains are considered carcinogenic and the functional intergenic fusion has therapeutic advantages. This report indicated that the combination of RET intergenic-breakpoint fusion and RET amplification may improve the response to pralsetinib therapy.

## Data availability statement

The datasets for this article are not publicly available due to concerns regarding participant/patient anonymity. Requests to access the datasets should be directed to the corresponding author.

## Ethics statement

Ethical review and approval was not required for the study on human participants in accordance with the local legislation and institutional requirements. Written informed consent was obtained from the patient for the publication of any potentially identifiable images or data included in this article.

## Author contributions

S-SW collected the patient data. FW drafted the manuscript. H-FQ conceptualized the study and contributed to writing—reviewing and editing. YH: supervision. All authors contributed to the article and approved the submission.

## Conflict of interest

Author H-HL and H-NW are employed by Acornmed Biotechnology Co., Ltd.

The remaining authors declare that the research was conducted in the absence of any commercial or financial relationships that could be construed as a potential conflict of interest.

## Publisher’s note

All claims expressed in this article are solely those of the authors and do not necessarily represent those of their affiliated organizations, or those of the publisher, the editors and the reviewers. Any product that may be evaluated in this article, or claim that may be made by its manufacturer, is not guaranteed or endorsed by the publisher.
