# Corrigendum: Case report: A novel intergenic *MIR4299/MIR8070-RET* fusion with RET amplification and clinical response to pralsetinib in a lung adenocarcinoma patient

**DOI:** 10.3389/fonc.2022.1113289

**Published:** 2022-12-13

**Authors:** Sha-Sha Wang, Fang Wang, Zhen Zeng, Fang Gao, Huan-Huan Liu, Hui-Na Wang, Yi Hu, Hai-Feng Qin

**Affiliations:** ^1^ Department of Oncology, Chinese People’s Liberation Army General Hospital, Beijing, China; ^2^ Department of Internal Medicine, OASIS International Hospital, Beijing, China; ^3^ Department of Medicine, Acornmed Biotechnology Co., Ltd., Beijing, China

**Keywords:** RET intergenic fusion, pralsetinib, lung adenocarcinoma, novel intergenic, tyrosine kinase inhibitors

In the published article, there were two errors made in the Case representation section regarding the dose of pralsetinib.

Firstly, a correction has been made to Case representation, Paragraph 1 on Page 2 of the article. The sentence “He was subsequently treated with pralsetinib (600 mg orally once daily)” has been corrected to “He was subsequently treated with pralsetinib (400 mg orally once daily)”.

Secondly, a correction has been made to Case representation, Paragraph 1 on Page 3. The sentence “Therefore, he was considered to achieve a partial response (PR) according to RECIST 1.1 criteria and continued to receive pralsetinib (600 mg orally once daily)” has been corrected to “Therefore, he was considered to achieve a partial response (PR) according to RECIST 1.1 criteria and continued to receive pralsetinib (400 mg orally once daily)”.

The authors apologize for these errors and state that they do not change the scientific conclusions of the article in any way. The original article has been updated.

