# Supplementary material for: Case Report: A novel intergenic MIR4299/MIR8070-RET fusion with RET amplification and clinical response to pralsetinib in a lung adenocarcinoma patient
Source: Front Oncol. 2022 Sep 26;12:929763. doi: 10.3389/fonc.2022.929763 (PMC9548572; doi:10.3389/fonc.2022.929763)
Supplement: Supplementary file 1 [file DataSheet_1.docx]

**Supplementary file**

**808 tumor-associated genes**

**Point mutations and insertions/deletions**

ABCB1 ABCC1 ABCC2 ABCC3 ABCC6 ABCC9 ABCG2 ABL1 ABL2 ACTB ACTG1 ACVR1 ACVR1B ACVR2A ADAMTS12 ADGRA2 ADGRG4 ADH1C AGO2 AKT1 AKT2 AKT3 ALDOC ALK ALOX12B AMER1 AMOT ANK2 ANKRD11 APC APCDD1 APOB APOE AR ARAF ARFRP1 ARID1A ARID1B ARID2 ARID5B ASB18 ASTN1 ASTN2 ASXL1 ASXL2 ASXL3 ATIC ATM ATP7A ATR ATRIP ATRX AURKA AURKB AXIN1 AXIN2 AXL B2M BABAM1 BACH1 BAP1 BARD1 BBC3 BCL10 BCL2 BCL2L1 BCL2L11 BCL2L2 BCL6 BCOR BCORL1 BCR BIRC3 BLM BMPR1A BORCS8- MEF2B BRAF BRCA1 BRCA2 BRD4 BRINP3 BRIP1 BTG1 BTK C14orf177 C6orf118 CA10 CALR CARD11 CARM1 CASP7 CASP8 CBFB CBL CBR1 CBR3 CCDC6 CCND1 CCND2 CCND3 CCNE1 CD274 CD276 CD74 CD79A CD79B CDA CDC42 CDC73 CDH1 CDH10 CDH12 CDH18 CDH9 CDK12 CDK4 CDK6 CDK8 CDKN1A CDKN1B CDKN2A CDKN2B CDKN2C CEBPA CENPA CFTR CHD2 CHD4 CHEK1 CHEK2 CHRM2 CHST3 CHUK CIC CNTNAP2 CNTNAP5 COL22A1 CRBN CREBBP CRKL CRLF2 CSDE1 CSF1R CSF3R CSMD3 CTCF CTLA4 CTNNA1 CTNNA2 CTNNB1 CUL3 CUL4A CUL4B CXCR4 CYBA CYLD CYP17A1 CYP2B6 CYP2C19 CYP2C8 CYP2C9 CYP2D6 CYP2E1 CYP3A4 CYSLTR2 DAXX DCAF12L1 DCAF12L2 DCAF4L2 DCK DCLRE1C DCUN1D1 DDB1 DDR2 DICER1 DIS3 DMD DNAJB1 DNMT1 DNMT3A DNMT3B DOT1L DPYD DROSHA DUSP27 DUSP4 E2F3 EED EGF EGFL7 EGFR EIF1AX EIF4A2 EIF4E ELF3 EML4 EMSY ENG EP300 EPAS1 EPCAM EPHA2 EPHA3 EPHA5 EPHA7 EPHB1 EPHX1 ERBB2 ERBB3 ERBB4 ERCC1 ERCC2 ERCC3 ERCC4 ERCC5 ERF ERG ERICH3 ERRFI1 ESR1 ESR2 ETV1 ETV4 ETV5 ETV6 EWSR1 EZH1 EZH2 EZR F3 FAM135B FAM175A FAM46C FAM58A FANCA FANCB FANCC FANCD2 FANCE FANCF FANCG FANCI FANCL FANCM FAS FAT1 FAT3 FBN2 FBXL7 FBXW7 FCGR3A FES FGD1 FGF10 FGF12 FGF14 FGF19 FGF23 FGF3 FGF4 FGF6 FGF7 FGFR1 FGFR2 FGFR3 FGFR4 FH FIP1L1 FLCN FLT1 FLT3 FLT4 FOLR3 FOXA1 FOXL2 FOXO1 FOXP1 FRK FRS2 FRYL FUBP1 FYN G6PC3 GABRA2 GABRA6 GALNT12 GAPDH GAST GATA1 GATA2 GATA3 GATA4 GATA6 GEN1 GGH GID4 GLI1 GNA11 GNA13 GNAQ GNAS GOPC GPR158 GPS2 GREM1 GRID1 GRIK3 GRIN2A GRM3 GRM8 GSK3B GSTA1 GSTM1 GSTM3 GSTP1 GSTT1 H3F3A H3F3AP4 H3F3B H3F3C HAPLN1 HCN1 HDAC9 HFE HGF HIST1H1C HIST1H2BD HIST1H3A HIST1H3B HIST1H3C HIST1H3D HIST1H3E HIST1H3F HIST1H3G HIST1H3H HIST1H3I HIST1H3J HIST2H3A HIST2H3C HIST2H3D HIST3H3 HLA-A HLA-B HNF1A HOXB13 HRAS HSD3B1 HSP90AA1 HTR1A ICOSLG ID3 IDH1 IDH2 IFNGR1 IFNL3 IGF1 IGF1R IGF2 IGFL3 IKBKE IKZF1 IL10 IL7R INHA INHBA INPP4A INPP4B INPPL1 INSR INSRR IQCJ IRF2 IRF4 IRS1 IRS2 ITPA JAK1 JAK2 JAK3 JUN KAT6A KCNA4 KCND2 KCNJ3 KCNT2 KDM5A KDM5C KDM6A KDR KEAP1 KEL KIF2B KIF5B KIT KLC1 KLF4 KLHL1 KLHL6 KMT2A KMT2B KMT2C KMT2D KMT5A KNSTRN KRAS LATS1 LATS2 LEPR LIG3 LIG4 LMO1 LOC349160 LPL LPPR4 LRFN5 LRIG3 LRP1B LRRC4C LRRIQ3 LRRK2 LRRTM4 LTK LYN LZTR1 MAD1L1 MAGI2 MALT1 MAP2K1 MAP2K2 MAP2K4 MAP3K1 MAP3K13 MAP3K14 MAP4K3 MAPK1 MAPK3 MAPKAP1 MAX MBD4 MCL1 MDC1 MDH2 MDM2 MDM4 MED12 MEF2B MEN1 MET MGA MITF MKRN3 MLH1 MLH3 MNAT1 MOCS2 MPL MRE11A MS4A3 MSH2 MSH3 MSH6 MSI1 MSI2 MST1 MST1R MTHFD1 MTHFR MTOR MUTYH MYB MYC MYCL MYCN MYD88 MYOD1 NAT2 NAV3 NBN NCAM1 NCOA3 NCOA4 NCOR1 NEGR1 NEIL1 NEIL3 NF1 NF2 NFE2L2 NFKBIA NKX2-1 NKX3-1 NLRP3 NLRP5 NOS3 NOTCH1 NOTCH2 NOTCH3 NOTCH4 NPM1 NQO1 NRAS NSD1 NTHL1 NTM NTRK1 NTRK2 NTRK3 NUF2 NUP93 NUTM1 PAK1 PAK3 PAK6 PAK7 PALB2 PALLD PAPPA2 PARK2 PARP1 PARP2 PARP3 PARP4 PAX5 PAX8 PBRM1 PCDH10 PCDH17 PDCD1 PDCD1LG2 PDGFRA PDGFRB PDHA2 PDK1 PDPK1 PDYN PDZRN3 PER1 PGR PHOX2B PIK3C2B PIK3C2G PIK3C3 PIK3CA PIK3CB PIK3CD PIK3CG PIK3R1 PIK3R2 PIK3R3 PIM1 PIP5K1A PLCG2 PLK2 PMAIP1 PML PMS1 PMS2 PNRC1 POLD1 POLDIP2 POLE POLM POM121L12 PPARD PPARG PPM1D PPP2R1A PPP4R2 PPP6C PRDM1 PRDM14 PREX2 PRIM2 PRKACA PRKAR1A PRKCI PRKD1 PRKDC PRSS1 PRSS8 PTCH1 PTEN PTP4A1 PTPN11 PTPRD PTPRS PTPRT PXDNL QKI RAB35 RAC1 RAC2 RAD21 RAD50 RAD51 RAD51B RAD51C RAD51D RAD52 RAD54L RAF1 RANBP2 RARA RASA1 RB1 RBBP8 RBM10 RECQL RECQL4 REG3A REL RET REV1 REV3L RFWD2 RHBDF2 RHEB RHOA RICTOR RINT1 RIT1 RNF43 ROCK1 ROS1 RP1L1 RPA1 RPL11 RPL35A RPL5 RPP30 RPS10 RPS17 RPS19 RPS24 RPS26 RPS6KA4 RPS6KB2 RPS7 RPTOR RRAGC RRAS RRAS2 RRM1 RRM2 RTEL1 RUNX1 RUNX1T1 RXRA RYBP RYR2 SALL1 SDC4 SDHA SDHAF2 SDHB SDHC SDHD SESN1 SESN2 SESN3 SETBP1 SETD2 SETMAR SF3B1 SH2B3 SH2D1A SHMT1 SHOC2 SHQ1 SLC14A2 SLC19A1 SLC22A2 SLC22A4 SLC34A2 SLC45A2 SLC8A1 SLCO1B1 SLCO1B3 SLIT2 SLIT3 SLITRK1 SLITRK2 SLITRK3 SLX4 SMAD2 SMAD3 SMAD4 SMARCA4 SMARCB1 SMARCD1 SMO SMYD3 SNCAIP SOCS1 SOS1 SOX10 SOX17 SOX2 SOX9 SPEN SPG7 SPHKAP SPINK1 SPOP SPRED1 SPTA1 SRC SRSF2 ST6GAL2 STAG2 STAT3 STAT4 STAT5A STAT5B STK11 STK19 STK40 STT3A SUFU SUZ12 SYK TAF1 TAP1 TAP2 TBP TBX3 TCEB1 TCF3 TCF7L2 TDG TEK TEKT4 TERC TERT TET1 TET2 TFE3 TFEB TG TGFB1 TGFBR1 TGFBR2 TGFBR3 TIPARP TLR4 TMEM127 TMPRSS2 TNFAIP3 TNFRSF14 TNFRSF17 TOP1 TOP2A TOPBP1 TP53 TP53BP1 TP63 TPM3 TPMT TRAF2 TRAF7 TRIM58 TRPC5 TRRAP TSC1 TSC2 TSHR TSHZ3 TYK2 TYMP TYMS U2AF1 UGT1A1 UGT1A8 UPF1 USP1 VEGFA VHL VTCN1 WHSC1 WHSC1L1 WISP3 WRN WT1 WWTR1 XIAP XIRP2 XPO1 XRCC1 XRCC2 XRCC3 YAP1 YES1 ZAN ZBTB2 ZFHX3 ZFHX4 ZIC1 ZIC4 ZIM2 ZNF217 ZNF423 ZNF521 ZNF536 ZNF703 ZNF804A ZNF804B ZNF831 ZRSR2

**Copy number variants (CNVs)**

ACVR1B AKT1 AKT2 AKT3 ALK AMER1 APC AR ARAF ARID1A ARID1B ARID2 ASXL1 ATM ATR ATRX AURKA AXIN1 B2M BAP1 BCL2 BCL2L11 BCOR BRAF BRCA1 BRCA2 BRD4 BTK CALR CARD11 CASP8 CBL CCND1 CCND2 CCND3 CCNE1 CD274 CDC73 CDH1 CDK12 CDK4 CDK6 CDKN1A CDKN1B CDKN2A CDKN2B CDKN2C CEBPA CHEK2 CIC CREBBP CRLF2 CSF1R CSF3R CTNNB1 CYLD DAXX DDR2 DNMT1 DNMT3A EGFR EP300 EPAS1 EPHA2 ERBB2 ERBB3 ERBB4 ERCC4 ESR1 EZH2 FANCA FANCC FBXW7 FGF3 FGFR1 FGFR2 FGFR3 FGFR4 FLT1 FLT3 FOXL2 FRK FUBP1 GATA1 GATA2 GATA3 GNA11 GNAQ GNAS H3F3A HGF HIST1H3B HNF1A HRAS IDH1 IDH2 IGF1R IGF2 IKZF1 IL7R JAK1 JAK2 JAK3 KDM5C KDM6A KDR KIT KLF4 KRAS LMO1 LRP1B MAP2K1 MAP2K2 MAP2K4 MAP3K1 MAPK1 MDM2 MDM4 MED12 MEN1 MET MLH1 MPL MSH2 MSH6 MTOR MYC MYCL Mycn MYD88 NCOA3 NCOR1 NF1 NF2 NFE2L2 NKX2-1 NOTCH1 NOTCH2 NPM1 NRAS NTRK1 NTRK2 NTRK3 PALB2 PARP1 PAX5 PBRM1 PDCD1LG2 PDGFRA PDGFRB PGR PIK3CA PIK3CB PIK3R1 PIK3R2 PLCG2 PMS2 POLD1 POLE PPP2R1A PRDM1 PTCH1 PTEN PTPN11 PTPRD RAC1 RAD51 RAF1 RB1 RICTOR RIT1 RNF43 ROCK1 RUNX1 SETBP1 SETD2 SF3B1 SMAD2 SMAD4 SMARCA4 SMARCB1 SMO SOCS1 SOX10 SOX9 SPOP SRSF2 STAG2 STK11 SYK TEKT4 TET2 TNFAIP3 TOP1 TOP2A TP53 TRAF7 TSC1 TSC2 TSHR TYK2 U2AF1 VEGFA VHL WT1

**Fusion variants**

ABL1 ALK AKT3 BCL2 BCR BRAF BRD4 EGFR ERBB4 ESR1 ETV1 ETV4 ETV5 ETV6 EWSR1 FGFR1 FGFR2 FGFR3 JAK2 KIT KMT2A MET MSH2 MYB MYC NOTCH2 NTRK1 NTRK2 NTRK3 PDGFRA RAF1 RARA RET ROS1 TFE3 TFEB TMPRSS2

**Germline variants**

ALK APC ATM ATR AXIN2 BAP1 BARD1 BLM BMPR1A BRCA1 BRCA2 BRIP1 CASP8 CDH1 CDKN2A CDKN2B CHEK2 CTLA4 ENG EPAS1 EPCAM FAM175A FANCA FANCB FANCC FANCE FANCD2 FANCG FAS FH FLCN GALNT12 GEN1 GPC3 GREM1 HFE HNF1B HOXB13 KIT KRAS MAX MDH2 MEN1 MET MITF MLH1 MLH3 MRE11A MSH2 MSH3 MSH6 MUTYH NBN NF1 NF2 NRAS NTHL1 PALB2 PALLD PBRM1 PDGFRA PHOX2B PMS2 POLD1 POLE PRKAR1A PRSS1 PTCH1 PTEN RAD50 RAD51C RAD51D RB1 RECQL4 RET RHBDF2 RINT1 RPL11 RPL35A RPL5 RPS10 RPS17 RPS19 RPS24 RPS26 RPS7 SDHA SDHAF2 SDHB SDHC SDHD SLX4 SMAD4 SMARCA4 SPINK1 STK11 TGFBR2 TMEM127 TP53 TSC1 TSC2 VHL WRN WT1 XRCC2 CDK12 CDK4 CHEK1 EGFR FANCI FANCL PPP2R2A RAD51B RAD54L
